# TORS as Part of Multilevel Surgery in OSA: The Importance of Careful Patient Selection and Outcomes

**DOI:** 10.3390/jcm11040990

**Published:** 2022-02-14

**Authors:** Peter M. Baptista, Natalia Diaz Zufiaurre, Octavio Garaycochea, Juan Manuel Alcalde Navarrete, Antonio Moffa, Lucrezia Giorgi, Manuele Casale, Carlos O’Connor-Reina, Guillermo Plaza

**Affiliations:** 1 Department of Otorhinolaryngology, Clinica Universidad de Navarra, Av. de Pío XII, 36, 31008 Pamplona, Spain; peterbaptista@gmail.com (P.M.B.); ndiazzu@unav.es (N.D.Z.); ogaraycoche@unav.es (O.G.); jalcalde@unav.es (J.M.A.N.); 2 School of Medicine, Campus Bio-Medico University, Via Alvaro del Portillo 21, 00128 Rome, Italy; l.giorgi@unicampus.it (L.G.); m.casale@unicampus.it (M.C.); 3 Integrated Therapies in Otolaryngology, Fondazione Policlinico Universitario Campus Bio-Medico, Via Alvaro del Portillo 200, 00128 Rome, Italy; 4 Otolaryngology Head and Neck Surgery, USP Hospital, Av. Severo Ochoa, 20, 29603 Marbella, Spain; coconnor@us.es; 5 Department of Otolaryngology, Hospital Universitario de Fuenlabrada, Cam. del Molino, 2, 28942 Fuenlabrada, Spain; guillermo.plaza@salud.madrid.org

**Keywords:** obstructive sleep apnea, robotic surgery, tongue base, multilevel collapse

## Abstract

Transoral robotic surgery (TORS) for Obstructive Sleep Apnea (OSA) is a relatively young technique principally devised for managing apneas in the tongue base area. This study summarizes and presents our personal experience with TORS for OSA treatment, with the aim to provide information regarding its safety, efficacy, and postoperative complications. A retrospective study was conducted on patients undergoing TORS with lingual tonsillectomy through the Da Vinci robot. The effectiveness of the surgical procedure was assessed employing the Epworth Sleepiness Scale (ESS) and overnight polysomnography with the Apnea-Hypopnea Index (AHI). A total of 57 patients were included. Eighteen patients (31.6%) had undergone previous surgery. The mean time of TORS procedure was 30 min. Base of tongue (BOT) management was associated with other procedures in all patients: pharyngoplasty (94%), tonsillectomy (66%), and septoplasty (58%). At 6 months follow-up visit, there was a significant improvement in AHI values (from 38.62 ± 20.36 to 24.33 ± 19.68) and ESS values (from 14.25 ± 3.97 to 8.25 ± 3.3). The surgical success rate was achieved in 35.5% of patients. The most frequent major complication was bleeding, with the need for operative intervention in three cases (5.3%). The most common minor complications were mild dehydration and pain. TORS for OSA treatment appears to be an effective and safe procedure for adequately selected patients looking for an alternative therapy to CPAP.

## 1. Introduction

Obstructive Sleep Apnea (OSA) is a prevalent disorder that affects up to 24% of adult men and 9% of adult women [1]. It is considered a severe social health problem that significantly increases cardiopulmonary and cerebrovascular morbidity, daytime sleepiness, poor work performance, and traffic accidents. OSA is an independent factor for hypertension, stroke, and myocardial infarction [2]. A multilevel collapse of the upper aerodigestive tract is the leading cause of OSA in most cases, causing repetitive partial and complete airway obstructions, intermittent hypoxemia, sympathetic nervous system output surges, and sleep arousals [3]. The retropalatal and retrolingual regions are the most frequent areas involved [4]. Continuous Positive Airway Pressure (CPAP) is considered the gold standard treatment for moderate to severe OSA. However, despite its proven effectiveness, a large percentage of patients are intolerant or reject its use [5]. Alternative therapeutic strategies are also available, including weight loss, positional therapy, oral appliances, myofunctional therapy, and surgical therapy. Several surgical procedures have been described for these situations. Since Vicini et al. [6] introduced the concept of Transoral Robotic Surgery (TORS) for the OSA treatment in 2010, many studies have been published regarding its efficacy [7]. In many ENT departments, TORS is nowadays considered a common surgical minimally approach for Base of Tongue (BOT) reduction in cases of OSA due to a lingual tonsil obstruction [8]. Furthermore, this procedure can be combined with other techniques, such as tonsillectomy, pharyngoplasty, genioglossal advancement, hyoid suspension, and many others in cases of a multilevel obstruction [9]. This study summarizes and presents our personal experience with TORS to manage OSA, whether as a standalone procedure or as a part of multilevel surgery. Our goal is to provide information regarding its safety, efficacy, and postoperative complications and to identify the outcome’s predictive factors.

## 2. Materials and Methods

### Patient Selection

A retrospective study was conducted at Department of Otorhinolaryngology at Clínica Universidad de Navarra (Pamplona, Navarra, Spain). From January 2011 to June 2021, 64 patients undergoing TORS with lingual tonsillectomy through the Da Vinci robot were included.

The procedure was either a standalone procedure or part of a multilevel operation, including pharyngeal, palatal, and/or nasal surgery. Nasal surgery included septoplasty and/or inferior turbinate reduction, endoscopic sinus surgery, and/or adenoidectomy. Palatal surgery included expansion sphincter pharyngoplasty or barbed reposition pharyngoplasty. Pharyngeal surgery included tonsillectomy. Tongue base surgery included lingual tonsillectomy, partial midline glossectomy, and epiglottoplasty. All procedures were performed by the same surgeon (PMB).

All patients underwent a complete ENT Physical exam, reporting awake BOT hypertrophy after Friedman’s Lingual tonsil hypertrophy [10], Epworth sleepiness scale (ESS), type I polysomnogram, and detailed examination in supine and left/right decubitus positions with Drug-Induced Sleep Endoscopy (DISE) with propofol, administered through target infusion pump to determine the level of obstruction according to European position paper on drug-induced sleep endoscopy [11], following VOTE classification [12]. All patients were counseled on possible alternative treatments and gave their consent to the procedure.

The selection criteria used for the indication of TORS surgery were:

1. Presence of symptomatic OSA (Epworth Sleepiness Scale (ESS) score > 11) and/or moderate to severe OSA (Apnea-Hypopnea Index (AHI) > 15).
2. Low tolerability or drop-out from CPAP (CPAP use less than 3 h per night).
3. Lingual tonsil hypertrophy (Friedman Type 3 or 4).
4. Adequate BOT exposure assessed during sleep endoscopy. Patients must have a minimum distance of 1.5 cm between the superior and inferior incisor teeth.
5. No contraindications to surgery (ASA score < 3, absence of micrognathia).

**Figure 1:**
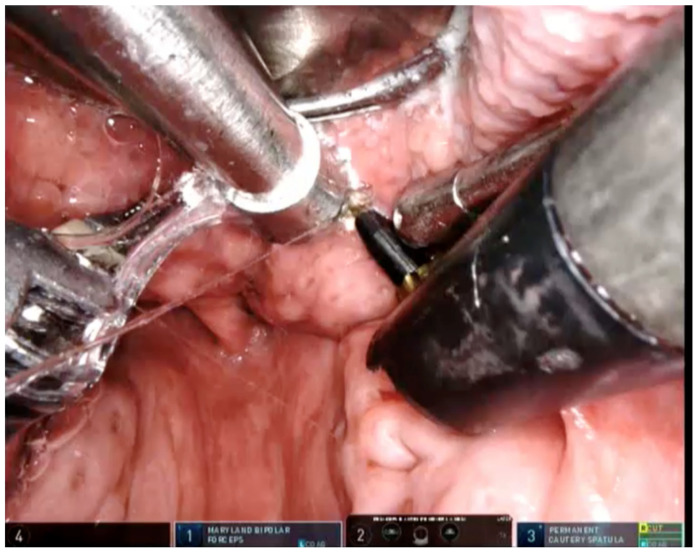
View of operative field before TORS: reduction of volume of tongue base and mouth aspirator, maryland dissector and bovie electrocautery.

**Figure 2:**
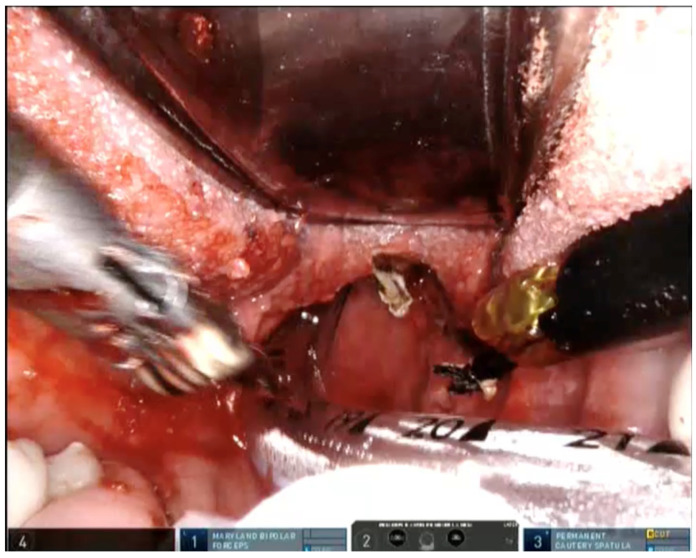
View of operative field after TORS: reduction of volume of tongue base and mouth aspirator, maryland dissector and bovie electrocautery.

Regarding TORS, in all the procedures, with the patient in the supine position, the tip of the tongue was fixed with a thick silk traction suture. A Storz Davis-Meyer mouth gag was used to obtain access and to visualize the lingual tonsil. BOT exposure was possible in all the cases. The robot was set up on the right side of the patient. Three Da Vinci robotic arms were used in the oral cavity, with the 30°-angled 3-dimensional endoscope in the center and the Maryland dissector in one arm. The second arm was the Monopolar cautery. The procedure began with a cut in the midline of the tongue base, from the foramen cecum to the vallecula. The incision was then extended laterally. In this way, it was possible to identify and preserve the neurovascular structures as the lingual artery and nerve. The right and left lingual tonsils were removed separately, with the right side followed by the left. An in-bloc resection of the lingual tonsil from superior to inferior and from medial to lateral was performed. We measured the volume of the tissue removed. Lingual tonsillectomy was always followed by epiglottoplasty. The epiglottis was held with the Maryland dissector and divided vertically along the midline 5 mm above the vallecula. A horizontal cut was then made in the right and the left portion to remove the upper one-third of the suprahyoid epiglottis (Figure 1 and Figure 2; Supplementary Materials).

The rate of immediate and delayed postoperative complications was also recorded. Complications were categorized as bleeding and other complications. Only patients with a minimum follow-up of 6 months were considered for surgical success assessment with ESS and a new type I polysomnogram. To report outcomes, Sher’s criteria were used to define success (50% reduction in AHI and an AHI less than 20% after surgery) [13].

A t-test was used to determine the difference between the AHI index and ESS before and after the procedure. A chi-squared test was used to determine the association between age, BMI, and AHI index, with surgical success according to Sher’s criteria. A value of *p* < 0.001 was regarded as being statistically significant. Quantitative data are shown as mean (SD), and qualitative data are represented as *n* (%). All statistical analyses were performed using IBM SPSS Statistics Visor.

## 3. Results

At the end of our selection process, 57 patients who satisfied the inclusion criteria were enrolled, 50 males (88%) and 7 females (12%). The mean age of the patients was 49.6 ± 12 years, and the mean BMI at the time of surgery was 28.8 ± 3.6 kg/m^2^. Demographic characteristics, pre-treatment and post-treatment average, and median values of AHI and ESS are summarized in Table 1.

All the subjects included suffered from moderate to severe OSA except four. In these patients, the indication was due to the lack of adherence or failure of non-surgical treatments. Eighteen patients (31.6%) had undergone previous surgery (septoplasty, turbinoplasty, tonsillectomy, palate surgery, adenoidectomy, or endoscopic sinus surgery). During TORS, the mean volume of BOT removed was 10 cc (6–15 cc). The mean total surgical time was 133 min, including all the other procedures included. The mean time of TORS procedure was 30 min. BOT management was associated with other procedures in all patients. The most common secondary procedures were pharyngoplasty (94%), tonsillectomy (66%), and septoplasty (58%). Table 2 shows all procedures performed with their frequencies.

All patients were admitted to the surgical intensive care unit (ICU) postoperatively. The median number of days in the ICU and hospital was 1 and 3 days, respectively. None of our patients underwent tracheostomy.

**Figure 3:**
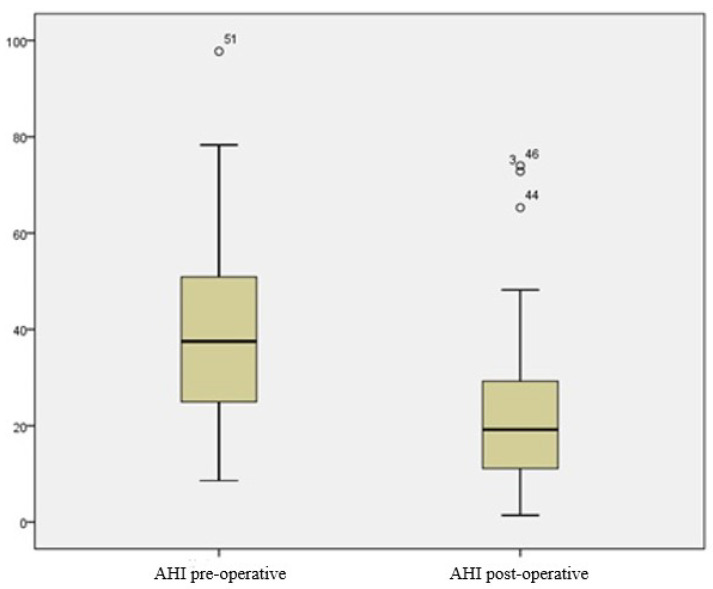
Pre-operative and post-operative AHI values. The central mark indicates the median, and the bottom and top edges of the box indicate the 25th and 75th percentiles, respectively. The whiskers extend to the most extreme data points not considered outliers, and the outliers are plotted individually using the ‘o’ marker symbol.

At the 6-month follow-up visit, there was a significant improvement in AHI values (from 38.62 ± 20, 36 events/h to 24.33 ± 19.68 events/h) and ESS values (from 14.25 ± 3.97 to 8.25 ± 3.3); (*p* < 0.001) (Figure 3). The surgical success rate was achieved in 35.5% of patients. In particular, we recorded the following AHI results: four patients with AHI < 15 events/h, four patients with AHI < 10 events/h, and three patients with AHI < 5 events/h. In five patients, there was a worsening of the AHI, and in four cases, the improvement was minimal.

There were a total of 10 complications in 9 patients (15.8%). Complications were classified as bleeding (8.8%) and other complications (8.8%), including atrial fibrillation, pulmonary thromboembolism, flap dehiscence, or rehospitalization for pain control. Table 3 outlines the complications that occurred.

The most frequent major complication was bleeding, with the need for operative intervention in three cases (5.3%). Bleeding was from the BOT in two cases and the tonsil in another case, and was controlled by transoral approach without the use of Da Vinci. The remaining two cases were self-limited bleeding, and the source could not be determined. Bleeding appeared in all cases between days 2 and 12 after the intervention.

The most common minor complications were mild dehydration and pain, although only two cases showed uncontrolled pain and required hospitalization for intravenous medications 5 and 10 days after the surgery. A few days later, the two patients were both discharged with no sequelae. No patient complained of impaired swallowing after the procedure after 2 weeks of surgery.

## 4. Discussion

OSA is an underestimated but severe health problem with a high social and economic impact. Since Vicini et al. described the application of TORS for BOT and epiglottis in OSA patients in 2010, many authors have obtained satisfactory results in different series of patients [6]. TORS is nowadays considered a common surgical procedure in cases of OSA due to a lingual tonsil obstruction.

In 2012, Friedman et al. described a 66.7% surgical success rate in a series of 40 patients [13]. In 2014, Toh et al. described a cure rate of 35% (AHI < 5 events/h) [3]. The latest systematic reviews and meta-analyses have shown a success rate between 48.2% and 68.4%, respectively, with the essential conditioning factors being a BMI <30 kg/m^2^ and its association with multilevel surgery as required [14]. Similarly, our study found a significant difference between pre- and post-operative AHI and the ESS values (*p* < 0.001). Moreover, more than one-third of the subjects (35.5%) achieved surgical success. Our study obtained a lower cure rate when compared to the results reported by Toh et al. [3], possibly because our sample was not homogeneous.

Our patients were subject to multilevel surgery. Therefore, our results are adequate as most patients had obstruction at diverse levels. In addition, some had poor prognostic features, such as BMI > 30 kg/m^2^ or AHI > 60 events/h. Unlike previous studies, significantly worse results have been reported in patients with high BMI and preoperative high AHI values [10]. It should be noted that in our group, the mean BMI or AHI between patients with surgical success and those without it (*p* = 0.8 and *p* = 0.18, respectively) was not statistically significant. Nonetheless, in all patients whose surgical procedure was considered successful, the AHI score was <60 events/h, and the mean value was lower than in the non-cured group (44.41 and 33.56, respectively). In addition, the mean age between cured patients and non-cured ones (*p* = 0.67) was not significant.

Although we did not compare this surgical technique with others, previous studies have compared TORS surgery to other therapeutic options. Cammaroto at al. compared TORS with Coblation Tongue Base Resection (CTBR) and concluded that complications occurred in 21.3% of the patients treated with TORS and in 8.4% of the patients treated with Coblation surgery [15]. On one hand, TORS seems to give slightly better results, allowing a broader surgical view and a measurable, more consistent removal of lingual tissue. On the other hand, in a randomized controlled trial comparing TORS with CTBR, the AHI improved from 29.7 ± 9 events/h to 10.7 ± 3.9 events/h (*p* < 0.001) following TORS, and from 27.2 ± 6.4 events/h to 10.3 ± 4 events/h in the Coblation group [16,17].

In a meta-analysis comprising 18 studies on TORS (834 patients) and 11 studies on CTBR (294 patients), it was observed that TORS allows a greater resection of the tongue base tissue compared to CTBR. The mean differences of AHI, ESS, and lowest oxygen saturation for TORS were −23.92, −7.6, and 5.83% (all *p* < 0.001). However, it was observed that the surgical success of the two is similar (57.6% vs. 60.3%, *p* = 0.4474), with a lower postoperative bleeding rate with TORS (3.3% vs. 7.5%, *p* = 0.0103), a longer operative time with TORS compared to CTBR (*p* > 0.0001), and a similar hospitalization time (*p* = 0.9047) [18,19].

Post-surgical bleeding that requires surgical revision has been described in 2.5% of cases after TORS, which was slightly lower than that commented in our group (8.8%). All bleeding cases occurred in our first 15 cases. Therefore, we can attribute this to a learning curve. Bleeding appeared in all cases between day 2 and 12 after the intervention.

Further complications with a 5 rate (8.8%) comprised arrhythmia, flap dehiscence, pulmonary thromboembolism, and rehospitalization for uncontrolled pain. Two patients required long-term anticoagulation therapy for atrial fibrillation and pulmonary embolism 4 and 15 days after the surgery, respectively. Nevertheless, the surgical procedure cannot be considered the leading cause of these complications because comorbidities such as obesity, hypertension, and dyslipidemia were previously present in both patients, and the anesthetic medication could have triggered these pathologies. The pulmonary embolism was diagnosed because the patient came to the emergency room, but the atrial fibrillation was discovered casually in an undiagnosed patient.

On the other hand, the higher rate of minor complications and the high costs of TORS must also be considered [10]. In the literature, the complications described have usually been rare and transient [2], with the most common being transient hypogeusia, transient pharyngeal oedema, and limited bleeding. In our study, dehydration and pain were the most common minor complications. In most cases, these complications were not severe and resolved with conservative measures.

Finally, regarding dysphagia after TORS, Eesa et al. [20] followed 78 patients operated by the group of Vicini for an average of 20 months (7–32 months). The results showed that dysphagia scales such as Anderson’s were not affected beyond the initial period. The mean time to begin with the oral diet was a single day, with a range from 1 to 3 days. None of the patients required a nasogastric tube. In our group, no patients complained of impaired swallowing longer than 2 weeks after the procedure.

We would like to point out that TORS was performed in conjunction with other level surgeries during the intervention, which shows that it is a safe procedure. However, there is a need for close mandatory vigilance in the first 24 h. Recently, Hypoglossal Nerve Stimulation (HNS) was introduced. It represents one of the latest surgical innovations in the OSA field, enhancing the upper airway neuromuscular tone to reduce collapsibility, which is thought to be the primary pathophysiological basis for OSA [21]. It allows an improvement of the airway by providing a stimulus on the genioglossus, geniohyoid, and palatoglossal muscles. On the other hand, TORS improves the airway by resection of the hyperplasic lingual tonsil, modifying the anatomical structures involved in the obstruction. These surgical procedures are not substitutes for each other but might be complementary. TORS is indicated if there is a very large lingual tonsil, but if there is a loss of tone of the tongue muscles, this should be addressed with HNS. Some papers have shown that HNS has many advantages, but significantly fewer complications, faster recovery, and better results [22,23]. In Spain, HNS is not covered by the Health System or insurance companies, and it represents an expensive treatment with a cost of approximately EUR 30.000 per patient. TORS is also costly and has its place, especially in the hospital where the surgeon works. It might be worth using, but most insurance companies do not cover it. Some patients may have to pay out of pocket for TORS, raising the surgery cost by EUR 2.000.

Our results emphasize that it is essential to select the patients adequately and exclude those with AHI > 60 events/h to achieve surgical success. Furthermore, it is also crucial to recommend weight loss in patients with a BMI > 30 kg/m^2^. Patients that do not fulfill these criteria should be excluded as surgical candidates. As for limitation of our study, it was a retrospective series, and our sample was not homogenous regarding the surgical procedure performed. Moreover, even though they were multilevel, performing different surgeries could have introduced a bias. It is hard to determine what percentage of reduction was due to TORS and what percentage was due to other surgeries. Finally, we must consider that the higher rate of complications we encountered may have been due to the surgeon’s learning curve, which is why most complications appeared in the first operated patients.

## 5. Conclusions

In patients with OSA due to retrolingual collapse accompanied by a hyperplasic lingual tonsil, confirmed by DISE, TORS is an effective measure in appropriately selected patients. It is a safe technique if performed by an experienced surgeon, with a reduced rate of complications if done correctly. Complications are infrequent and transitory. Post-operative surveillance in an intensive care unit is critical to ensure the control of possible adverse events.

## Figures and Tables

**Table 1:** Subject demographic characteristics.

	Mean	SD
Age	49.63	12.09
BMI	28.84	3.66
AHI Pre	38.62	20.36
ESS Pre	14.25	3.97
AHI Post	24.33	19.68
ESS Post	8.25	3.3

SD: Standart Deviation; BMI: Body Mass Index; EES: Epworth Sleepiness Scale; AHI: Apnea-hypopnea Index.

**Table 2:** Secondary procedures associated with TORS.

Intervention	*n* (%)
Septoplasty	33 (58%)
Turbinoplasty	32 (56%)
Adenoidectomy	3 (5%)
Tonsillectomy	38 (66%)
Pharingoplasty	54 (94%)
Epigotoplasty	28 (49%)
Nasal Endoscopic Surgery	2 (3%)

**Table 3:** Secondary procedures associated to TORS.

Complication	*n* (%)
No complications	48 (84%)
Bleeding	5 (8.8%)
Other Complications *	9 (16%)

* Atrial fibrillation, pulmonary thromboembolism, flap dehiscence, and rehospitalization for pain control.

## Data Availability

The data presented in this study are available on request from the corresponding author. The data are not publicly available due to originality of the work.

## Supplementary Materials

The following supporting information can be downloaded at: https://www.mdpi.com/article/10.3390/jcm11040990/s1, Video S1: Da Vinci Robotic lingual tonsil resection.
